# Does Anxiety Cause Freezing of Gait in Parkinson's Disease?

**DOI:** 10.1371/journal.pone.0106561

**Published:** 2014-09-24

**Authors:** Kaylena A. Ehgoetz Martens, Colin G. Ellard, Quincy J. Almeida

**Affiliations:** 1 Sun Life Financial Movement Disorders Research and Rehabilitation Centre, Wilfrid Laurier University, Waterloo, Ontario, Canada; 2 Cognitive Neuroscience, Department of Psychology, University of Waterloo, Waterloo, Ontario, Canada; University of California, Merced, United States of America

## Abstract

Individuals with Parkinson's disease (PD) commonly experience freezing of gait under time constraints, in narrow spaces, and in the dark. One commonality between these different situations is that they may all provoke anxiety, yet anxiety has never been directly examined as a cause of FOG. In this study, virtual reality was used to induce anxiety and evaluate whether it directly causes FOG. Fourteen patients with PD and freezing of gait (Freezers) and 17 PD without freezing of gait (Non-Freezers) were instructed to walk in two virtual environments: (i) across a plank that was located on the ground (LOW), (ii) across a plank above a deep pit (HIGH). Multiple synchronized motion capture cameras updated participants' movement through the virtual environment in real-time, while their gait was recorded. Anxiety levels were evaluated after each trial using self-assessment manikins. Freezers performed the experiment on two separate occasions (in their ON and OFF state). Freezers reported higher levels of anxiety compared to Non-Freezers (p<0.001) and all patients reported greater levels of anxiety when walking across the HIGH plank compared to the LOW (p<0.001). Freezers experienced significantly more freezing of gait episodes (p = 0.013) and spent a significantly greater percentage of each trial frozen (p = 0.005) when crossing the HIGH plank. This finding was even more pronounced when comparing Freezers in their OFF state. Freezers also had greater step length variability in the HIGH compared to the LOW condition, while the step length variability in Non-Freezers did not change. In conclusion, this was the first study to directly compare freezing of gait in anxious and non-anxious situations. These results present strong evidence that anxiety is an important mechanism underlying freezing of gait and supports the notion that the limbic system may have a profound contribution to freezing in PD.

## Introduction

Freezing of gait (FOG) is arguably the most debilitating symptom of Parkinson's disease (PD) and commonly occurs in confined spaces (such as doorways, and corridors) [1], [2], under time constraints (such as entering an elevator, or rushing to answer a phone) [1], [3], [4] and in the dark [5]. Interestingly, one link between these very different situations is that they all may provoke anxiety. It has been speculated that anxiety might trigger freezing of gait [1],[4],[6],[7]. In fact a recent study showed that significantly greater amounts of freezing were found when participants walked in complete darkness toward a doorframe compared to complete darkness into open space [5]. Based on these findings, it was hypothesized that anxiety might play an important role in triggering freezing behaviour; however, no study to date has gone beyond correlational analyses to test directly whether anxiety might be a cause of freezing.

Anxiety is a not only a common non-motor symptom of Parkinson's disease (affecting up to 69% of patients [8], [9]), but it is also one of the most influential predictors of quality of life in those with PD [10], [11]. Several studies have shown that anxiety is associated with more severe gait disturbance in PD (PIGD subtype) [12], [13]. Interestingly, a higher prevalence of anxiety and other mood disorders have been reported amongst the specific subgroup of patients that experience freezing of gait [12]. Moreover, panic attacks have been reported prior to and during freezing of gait episodes [6]. Physiological measures such as heart rate support this association between anxiety and freezing, since heart rate increases have been reported just prior to and during a freezing episode [7]. Taken together, there are several lines of research that suggest stress and anxiety are not only related, but might play a key role in the underlying mechanism of freezing of gait.

Although the pathophysiology of freezing of gait remains unclear, increasing evidence suggests that non-motor systems are likely involved in its underlying mechanism [7], [14]. Of all the recent hypotheses attempting to explain freezing of gait (for complete review see [15]), one specific model (cross-talk model) has emphasized the potential role of the limbic system. According to the model, striatal dopaminergic loss in Parkinsonian conditions can be compounded by competing inputs from the cognitive, limbic and motor loops, which in certain situations can overload the striatum's processing capacity, thereby leading to freezing of gait [16]. Based on this hypothesis, one might expect that in anxious situations, the increased “limbic load” could in fact elicit freezing of gait.

The cross talk model not only suggests that the limbic system might play an important role in freezing of gait but also emphasizes the role of striatal dopamine in integrative basal ganglia processes, such that insufficient levels may result in a functional deficit [16]. Interestingly, a growing body of evidence suggests that freezing of gait is more severe in the OFF state and improves with dopaminergic medication [17]–[19]. However, there is little consensus as to how dopamine contributes to the underlying mechanism of freezing and how it may act to ameliorate freezing severity and behaviour. Many patients report greater levels of anxiety during their off-period, and some researchers have suggested that this may represent a dopaminergic “mood-off” phenomenon [12], [20]. However, this has also been debated since other researchers have shown that levodopa exacerbates anxiety symptoms [13], [21], [22]. The nucleus accumbens is central to processing and integrating emotional (limbic) information in the basal ganglia and is mediated by dopaminergic input [23]. Therefore, freezing of gait might be expected to be greater in the OFF state, especially when walking in an anxiety-provoking environment since this situation would create an overload of information to be processed by a “dopamine depleted” basal ganglia. However, with dopaminergic replacement therapy (ON state), there may be more integrated processing across the basal ganglia resulting in less freezing of gait compared to the OFF state.

The current study is the first to utilize virtual reality to induce anxiety and directly measure freezing of gait while walking, in order to establish whether anxiety causes freezing of gait in Parkinson's disease. Virtual reality has been shown to be an effective tool to immerse participants in specific situations in order to induce freezing-like behaviour, as well as the typically associated step-to-step variability changes [17], [24] that have been identified in real-life gait studies of freezing. The secondary aim of this study was to investigate whether dopaminergic medication influences freezing of gait in anxious situations. To achieve these aims, we asked participants who experience freezing of gait to perform the experimental protocol on two separate occasions: once ON and once OFF regular dopaminergic medication to determine whether the lack of dopamine exacerbated freezing of gait in anxious environments.

## Materials and Methods

### Participants

Thirty-one participants with Parkinson's disease were tested in this study. Table 1 shows the demographic characteristics and clinical details of participants. All participants were recruited through the Sun Life Financial Movement Disorder Research and Rehabilitation Centre database at Wilfrid Laurier University in Waterloo, Canada. Fourteen patients were confirmed to experience freezing of gait using the previously established criteria: (i) previous diagnosis of idiopathic Parkinson's disease by a neurologist and a history of freezing of gait; (ii) patients self-reported freezing of gait using UPDRS-II; (iii) a movement disorder specialist confirmed the presence of FOG during assessment prior to participation in the study (see [2], [25] for full procedure). Participants were excluded if they could not walk 10 m unassisted, had vertigo, motion sickness, severe kyphosis, other neurological disorders, severe head tremor or dyskinesias (since it would make the virtual environment appear to be shaking, increasing the difficulty and likelihood of motion sickness). Patient files were also carefully screened for co-morbid conditions (i.e. history of stroke, visual impairments, hearing loss, peripheral neuropathies, or diabetes). The Unified Parkinson's Disease Rating Scale motor section (UPDRS-III) [26] was administered by a certified clinician and assessed disease severity, while the Modified Mini Mental State Exam (3MS) [27] screened for dementia. Additionally, all participants completed the State and Trait Anxiety Inventory [28] assessing baseline levels of anxiety prior to completing the experiment; Geriatric Depression Scale [29]; and the SCOPA-AUT questionnaire which has been shown to assess the integrity of the autonomic nervous system [30]. Finally, a simulator sickness questionnaire was completed once before the experiment and then again after the experimental walking trials to quantify any adverse effects as a result of the virtual reality protocol.

**Table 1:** Demographic characteristics and clinical details of participants.

	Freezers	Non-freezers	P-value
Number	14	17	
Age	71 (7.8)	66 (8.7)	p = 0.13
Gender	3 F	3 F	
Symptom Severity (UPDRS-III)	34 (10.1)	20 (10.4)	p = 0.0009
3MS	95 (7)	96 (4.5)	p = 0.53
Dosage (LED)	204.1 (62.7)	223.1 (98.9)	p = 0.54
STAI-Trait	33 (6.9)	32 (6.6)	p = 0.74
STAI-State	34 (8.7)	30 (5.9)	p = 0.19
GDS	7 (3.4)	7 (5)	p = 0.82
SCOPA-AUT	16 (5.6)	16 (4.4)	p = 0.89
Pre-SSQ	6 (4.7)	9 (7.1)	p = 0.47
Post-SSQ	9 (7.4)	8 (5.5)	p = 0.97
	**‘OFF’ Freezers**	**‘ON’ Freezers**	**P-value**
UPDRS-III	39 (10.6)	32 (11.3)	p = 0.0001
STAI-Trait	35 (8.9)	33 (5.8)	p = 0.37
STAI-State	37 (10.8)	31 (8.9)	p = 0.20

3MS: Modified Mini Mental State Exam; STAI: State-Trait Anxiety Inventory; GDS: Geriatric Depression Scale; SSQ: Simulator Sickness Questionnaire.

### Apparatus

Participants were outfitted in a completely wireless virtual reality (VR) head mounted display (HMD) system that was tracked in real-time using three infrared light emitting diodes attached to a rigid body which was secured to the virtual reality helmet. The viewpoint in the virtual environment was controlled by the position and movement of the rigid body captured by seven OPTOTRAK Certus cameras (NDI Principles Inc., Waterloo, Canada). This synchronized the participants' position and movements, allowing the viewpoint to update in real-time, creating an immersive virtual setting.

The virtual environment used in this study was constructed using virtual reality software, *Vizard* (Worldviz L.L.C., Santa Barbara, USA). The testing environment was delivered using a high definition, low latency wireless link to a zSight head mounted display (HMD) (Sensics Inc., Columbia, USA) that featured a 60-degree field-of-view with 100% binocular overlap and a 1280×1024 full-colour pixels per eye resolution. The HMD also had a light-blocking cover that was pressed firmly to the participants' face, which prevented participants from seeing the real-world environment around them and allowed them to focus only on the virtual environment. In order to make the virtual environments as immersive as possible, the experiment was completed in a dark room which prevented participants from seeing the floor, their own feet, or the spotters' feet walking beside them in the “real-world”.

The visual focus and eye width settings were adjusted for each participant at the beginning of the study and confirmed to display a clear stereoscopic 3-D image. Participants were positioned at the end of the laboratory room and a GAITRite carpet (over 8 m in length) was located on the floor in front of them lengthwise. The GAITRite carpet (CIR systems Inc., Sparta, USA) was used to capture spatiotemporal aspects of gait during each walking trial.

### Experimental design and paradigm

**Figure 1:**
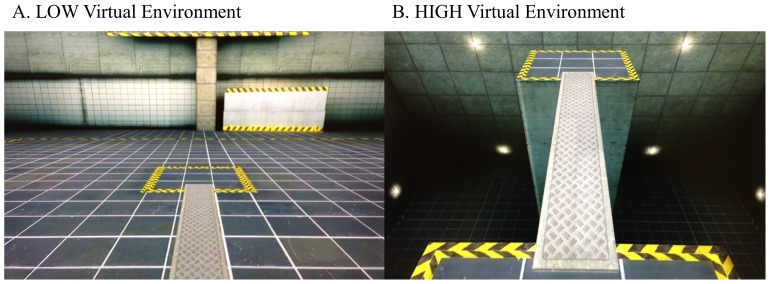
The experimental paradigm. Patients walked across the virtual plank in two virtual environments: A) LOW: while the plank was located on the ground; B) HIGH: participants viewed the floor descend, and then were instructed to walk across the plank above the deep pit.

All participants completed a total of 10 randomized walking trials in two different conditions. To begin, they stood on the edge of a GAITRite carpet which was calibrated to visually display the starting platform in virtual reality. To complete the task, participants were required to walk across a plank (6 m in length ×1 m in width) to the opposite platform in one of two 3-D virtual environments (*Vizard*, Worldviz L.L.P., Santa Barbara, USA) (see Figure 1). In the LOW condition, all participants were required to walk across a plank that was located on the floor of the virtual environment (Figure 1A). In contrast, during the HIGH condition, all participants viewed the floor surrounding the platform as it descended creating a deep pit below. Participants were required to walk across the plank which appeared to be approximately 8 m above the deep pit (Figure 1B). After walking across the plank to the opposite platform in each trial, a 9 point self-assessment manikin scale [31] would be displayed and patients were asked to rate their feelings of stress and anxiety using the self-assessment manikins. Once an anxiety rating was given, the head mounted display would present a black screen and a research volunteer would guide the patient back to the start position for the next trial. A standing rest period of 30 seconds was given after each trial to prevent carry-over effects from anxiety on the previous trial.

Our primary research question was whether anxiety influences freezing of gait. Thus, it was most ecologically valid to test all of the participants in the ON state since this is typically their medication state during their daily activities [5]. All patients were tested approximately one hour after taking their regular dosage of anti-Parkinsonian medication. However, since there has been debate as to whether dopaminergic medication reduces or exacerbates anxiety in Parkinson's disease, and furthermore it is controversial whether freezing of gait is dopa-responsive, all patients with freezing of gait were invited to be tested in both medication states on two separate occasions (counterbalanced across participants). Of the fourteen Freezers, ten gave consent to complete this study twice (once OFF and once ON their regular dopaminergic medication), however one patient was unable to complete any walking trials in the HIGH condition due to severe akinetic freezing in the OFF state, and another two patients dropped out after completing the study once in their ON state, convinced that they would not be able to perform any trials in their OFF state. Thus, seven patients with freezing completed this study once after at least a 12 hour withdrawal from dopaminergic medication overnight (this withdrawal was increased to 24 hours for dopamine agonists) and again approximately one hour after their regular dosage. Ethical approval was obtained by both the Research Ethics Board at Wilfrid Laurier University as well as the Office of Research Ethics at the University of Waterloo. Written informed consent was obtained from all participants before participating according to the Declaration of Helsinki.

### Data analysis

The primary outcome measure was the percent of each trial spent frozen since it is known to be the most reliable measure of freezing of gait [32]. The number of freezing of gait episodes was also recorded and compared. A freezing of gait episode was defined both objectively and subjectively as suggested in previous research [5], [18]. First, trials with freezing of gait were visually identified through video playback of steps recorded on the GAITRite carpet using PKMAS software (Protokinetics, Havertown, USA). If freezing of gait was observed, each step during the trial was exported and analyzed. A freezing of gait episode was defined as any period where the stride velocity dropped between zero (i.e. completely stopped) and one standard deviation above zero of their regular velocity on that trial. Previous studies have used this criterion [5], [33] since it is a stringent, objective measure of FOG. This procedure allowed us to quantify the number of freezing of gait episodes, the duration of each freezing episode and calculate the percent of each trial spent frozen.

Previous research has found that spatial and temporal aspects of gait, such as step-to-step variability, can be indicative of an upcoming FOG occurrence [34]–[36]. Furthermore, since freezing of gait is difficult to evoke in experimental settings, it is also important to understand changes in gait behaviour that may not result in a full blown freezing episode in response to the experimental manipulations. For these reasons, we chose to also analyse participants' gait characteristics such as velocity (cm/s), mean step length (cm), step length variability (Coefficient of Variation – CV), mean step width (cm), step width variability (CV), step time (s) and step time variability (CV), which tend to be indicative of cautious walking in response to anxiety. It should be noted that any freezing of gait episodes detected were removed from the secondary gait analysis to avoid bias comparison between groups and conditions. The dependent gait variables were analyzed using PKMAS software.

### Statistical methods

Baseline demographic variables were compared between groups and also within the Freezer subgroup between medication states using independent and dependent t-tests. Assumptions were assessed and when necessary (i.e. Mauchly's test of sphericity was violated) then the degrees of freedom were corrected using Greenhouse-Geisser estimate of sphericity and reported. A mixed repeated measures ANOVA (group x condition x trial) was used to evaluate changes in the anxiety self-assessment ratings and gait variables across all participants. The frequency of FOG episodes, the total duration of time spent frozen, and the percent of each trial spent frozen were analyzed using a repeated measures ANOVA with 2 factors of repeated measures (i.e. condition and trial), allowing for a comparison of the FOG variables between the two conditions specifically within the freezer group. In all cases, Tukey's HSD post hoc procedure was used to further investigate significant differences.

Since very few participants were able to complete the study both ON and OFF their dopaminergic medication, and FOG did not occur in any individuals during the LOW condition while ON their dopaminergic medication (causing a lack of variance); none of the freezing of gait variables were statistically compared between medication states. These variables were still calculated and reported (see Table 2). In order to evaluate dopaminergic influences on anxiety, repeated measures ANOVA (medication x condition x trial) were used to compare anxiety ratings.

**Table 2:** Freezing of gait measures during walking in virtual reality.

	Freezers (ON N = 14)			Freezers (OFF N = 7)		Freezers (ON N = 7)	
	Low	High	p-value	Low	High	Low	High
Percent of Each Trial Spent Frozen	11.03 (23)	23.1 (28.7)	p = 0.005	2.09 (3.8)	81.6 (123.8)	0	51.88 (65.1)
Total Number of Freezing Episodes	88	231	p = 0.013	3	111	0	26
Average Number of Freezing Episodes per trial	1.3 (2.6)	3.3 (5.3)	p = 0.013	0.4 (0.8)	15.9 (30.2)	0	3.8 (3.4)
Average Duration of Each Freezing Episode (sec)	1.31 (4.0)	3.04 (6.8)	p = 0.14	0.58 (1.2)	7.5 (10)	0	7.91 (12.4)

## Results

### Baseline data

Results showed that Freezers had significantly higher motor symptom severity (UPDRS-III) compared to the Non-Freezers (t(29) = 3.71, p = 0.0009). Importantly, these groups were not statistically different at baseline on the following demographic measures (See Table 1); age (t(29) = 1.54, p = 0.13), levels of Trait anxiety (t(29) = 0.34, p = 0.74), State anxiety (t(29) = 1.35, p = 0.19), Depression (t(28) = 0.23, p = 0.82), SCOPA-AUT (t(27) = 0.14, p = 0.88), pre-simulator sickness (t(28) = 0.73, p = 0.47), and post-simulator sickness questionnaire (t(29) = 0.04, p = 0.97). It is important to note however that the Freezers had a lower cognitive status compared to Non-Freezers (t(28) = 0.64, p = 0.053), although this was not quite significant.

### Anxiety ratings

**Figure 2:**
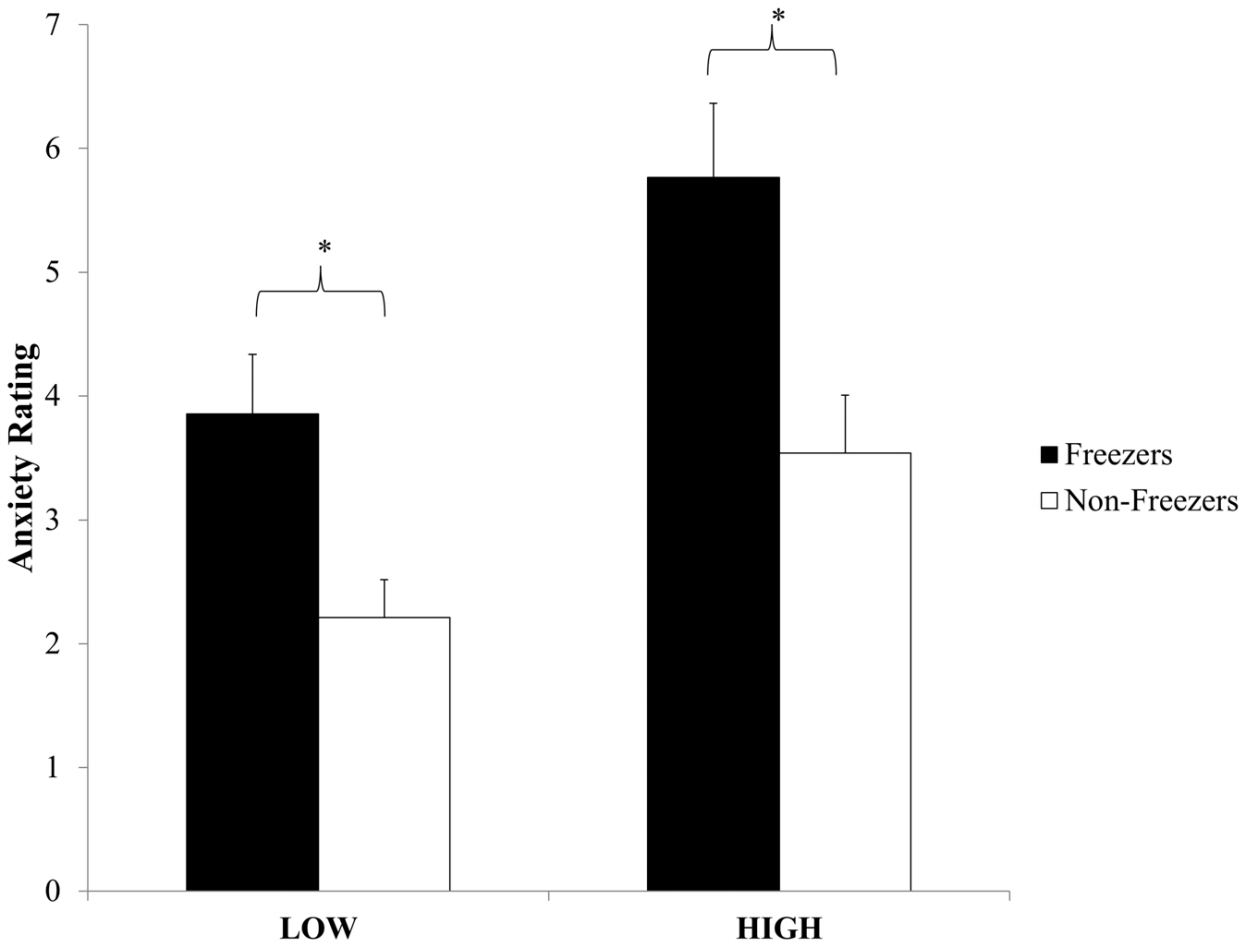
Comparison of anxiety ratings after participants walked across the plank. Error bars represent standard error of the mean. * illustrates significant differences between groups (p<0.05).

A main effect of group (F(1,29) = 16.96, p = 0.0003) showed that Freezers reported higher levels of anxiety during the experiment compared to Non-freezers. A main effect of condition (F(1,29) = 29.83, p<0.0001) revealed that all participants reported higher levels of anxiety during the HIGH condition compared to the LOW (Figure 2). Finally, a main effect of trial (Greenhouse-Geisser correction: F(2.3, 67.9) = 8.88, p<0.0001) demonstrated that participants reported the greatest amount of anxiety on the first trial in each condition.

### Influence of medication state on anxiety ratings

Symptom severity was significantly greater when Freezers were tested in their OFF state compared to ON their regular dopaminergic medication (t(6) = 8.48, p = 0.0001). Results also showed that baseline levels of anxiety (both trait and state) prior to the experiment did not change significantly between medication states (t(6) = 0.97, p = 0.36; t(6) = 1.44, p = 0.20) (Table 1). Finally, there was no main effect of medication state on anxiety ratings during the walking trials (F(1,6) = 0.12, p = 0.92).

### Freezing of gait measures

#### Percent of Each Trial Spent Frozen

**Figure 3:**
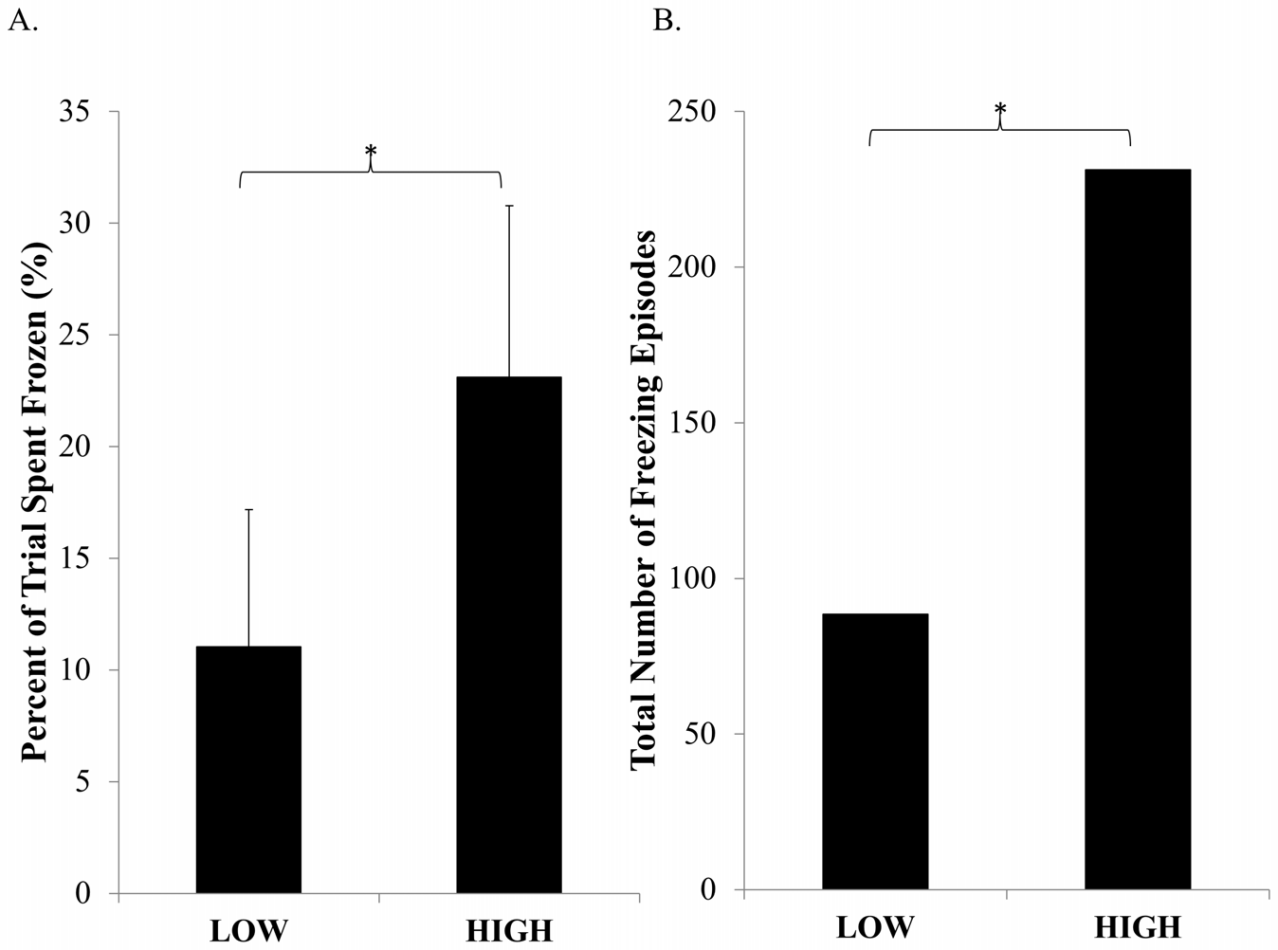
Comparison of on-state freezing of gait between HIGH and LOW conditions. Error bars represent standard error of the mean. * illustrates a significant difference between conditions within Freezers (p<0.05).

Freezers spent a significantly greater percent of each trial frozen during the HIGH condition compared to LOW (F(1,13) = 11.35, p = 0.005) (see Figure 3A). There was no main effect of trial found (F(4,52) = 0.49, p = 0.74).

#### Frequency of Freezing of Gait

Freezers experienced significantly greater number of freezing episodes during the HIGH condition compared to the LOW (F(1,13) = 8.29, p = 0.013) (see Figure 3B). There was no main effect of trial found (F(4,52) = 0.53, p = 0.72).

#### Duration of Freezing Episodes

There were no significant main effects found for either condition (F(1,13) = 2.48, p = 0.14) or trial (F(4,52) = 2.11, p = 0.09), suggesting that the duration of each freezing episodes was similar regardless of anxiety condition or trial.

### Gait parameters

#### Velocity

Overall, Freezers walked significantly slower than Non-Freezers (F(1,29) = 25.31, p<0.0001) (See Table 3), although all participants walked significantly slower during the HIGH condition compared to the LOW (F(1,29) = 53.54, p<0.0001). A significant condition by trial interaction (Greenhouse-Geisser estimate: F(2.6, 76) = 3.36, p = 0.028) showed that all participants reduced their velocity more during the first trial of the HIGH condition compared to the LOW, although as the trials progress the participants' increased their velocity in both conditions.

**Table 3:** Comparison of overall spatiotemporal aspects of gait between Freezers and Non-freezers.

Spatiotemporal variables	Freezers (N = 14)		Non-Freezers (N = 17)		p-value
	Low	High	Low	High	Group Differences
Velocity (cm/s)	42.44 (23)	28.67 (19.3)	81.8 (23.3)	67.29 (27.4)	p<0.0001
Step length	21.99 (12)	14.62 (9.3)	48.92 (11.7)	39.34 (14.5)	p<0.0001
Step time	0.58 (0.4)	0.68 (0.4)	0.60 (0.08)	0.60 (0.1)	p = 0.65
Step Width	13.72 (3.3)	13.47 (3.2)	10.94 (4.3)	10.8 (4.2)	p = 0.047
Step length CV (%)	45.06 (46.1)	70.88 (77.8)	11.24 (4.7)	18.71 (11.5)	p = 0.002
Step time CV (%)	22.92 (25.2)	29.94 (32.9)	8.24 (4.5)	12.74 (9.3)	p = 0.009
Step width CV (%)	14.55 (7)	14.18 (8.3)	27.64 (29)	25.78 (19.7)	p = 0.013

CV: Coefficient of variation.

#### Step Length

Overall, Freezers walked with a significantly short step length (F(1,29) = 43.05, p<0.0001) compared to Non-Freezers. Moreover, during the HIGH condition, all participants walked with a reduced step length compared to the LOW condition (Greenhouse-Geisser estimate: F(2.6, 75.5) = 31.55, p<0.0001). A group by trial interaction (F(4,116) = 3.7, p = 0.007) revealed that Freezers had significantly shorter steps during the first trial (regardless of condition) compared to the third trial, whereas Non-Freezers increased their step length significantly by the second trial (compared to the first) and had even greater step length by the final trial (compared to the second). A significant interaction between condition and trial (Greenhouse-Geisser estimate: F(3,85.6) = 6.52, p<0.0001) showed that all participants had a greater reduction in step length during the first trial of the HIGH condition compared to the LOW. In both conditions, participants' step length improved as the trials progressed.

#### Step Time

There were no significant main effects of group (F(1,29) = 0.22, p = 0.65), condition (F(1,29) = 0.96, p = 0.33), or trial (F(1.2, 35.7) = 2.61, p = 0.1) for step time.

#### Step Width

Overall, Freezers walked with a significantly smaller step width compared to Non-Freezers regardless of condition (F(1,29) = 4.32, p = 0.047). There were no significant effects of condition (F(1,29) = 1.16, p = 0.29) or trial (F(4,116) = 1.76, p = 0.14).

#### Step Length Variability

**Figure 4:**
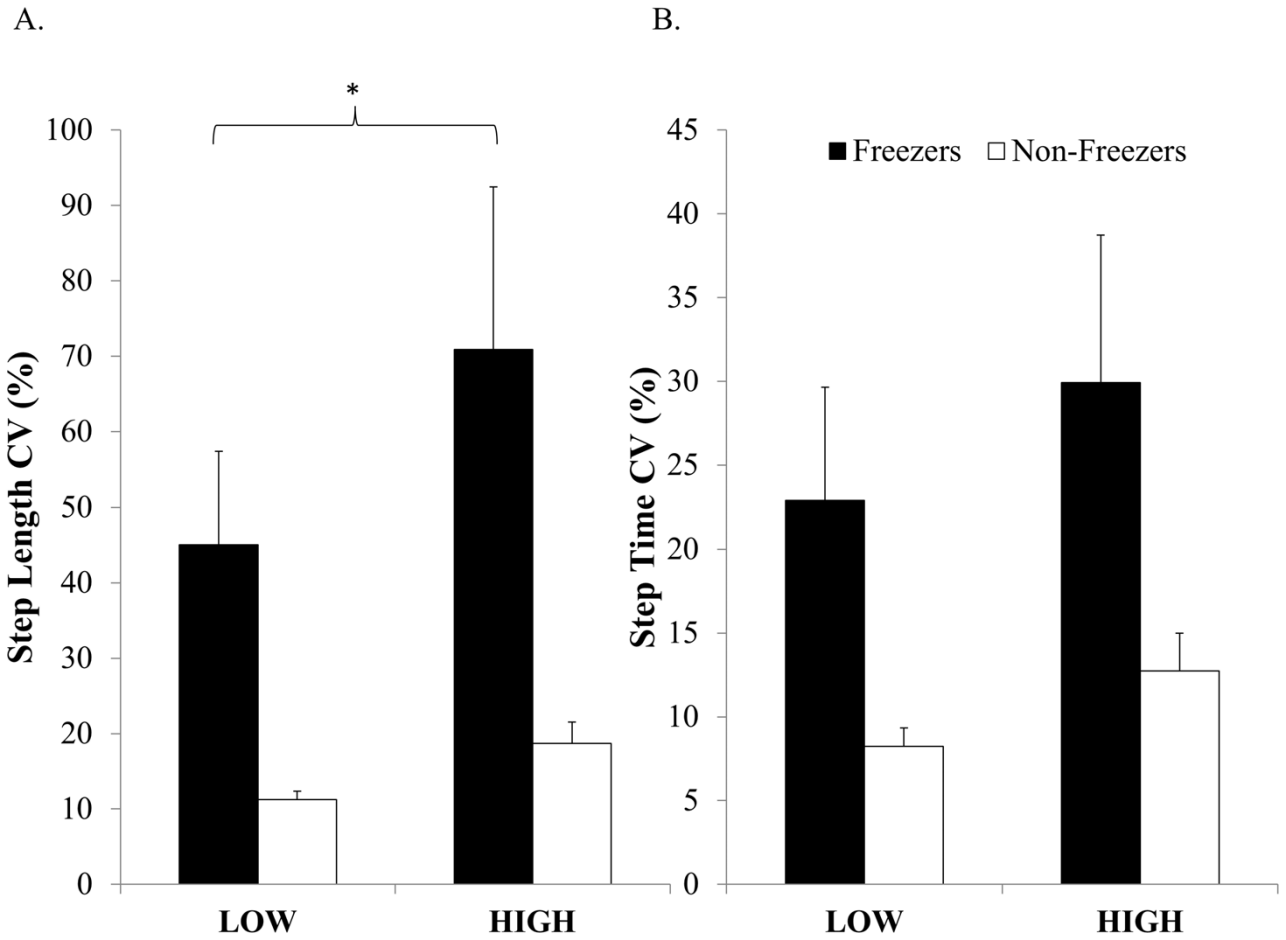
Comparison of step-to-step variability between Freezers and Non-Freezers. Error bars represent standard error of the mean. * illustrates a significant difference within Freezers between the HIGH and LOW condition (p<0.05).

Freezers had higher step length variability compared to Non-Freezers (F(1,29) = 11.62, p = 0.002), although all participants walked with a higher step length variability during the HIGH condition compared to the LOW (F(1,29) = 12.8, p = 0.001). Interestingly, there was a near significant interaction between group and condition (F(1,29) = 3.88, p = 0.058). Tukey's post hoc showed that Freezers had similar step length variability during the LOW condition as Non-Freezers, however Freezers had significantly greater variability during the HIGH condition compared to Non-Freezers (p = 0.002) (see Figure 4A).

#### Step Time Variability

Freezers had higher step time variability compared to Non-Freezers (F(1,29) = 7.79, p = 0.009), although all participants walked with a higher step time variability during the HIGH condition compared to the LOW (F(1,29) = 13.9, p = 0.0008) (see Figure 4B).

#### Step Width Variability

Overall, Freezers walked with a significantly lower step width variability compared to Non-Freezers regardless of condition (F(1,29) = 7.04, p = 0.013). There were no significant effects of condition (F(1,29) = 0.43, p = 0.52) or trial (F(4,116) = 0.32, p = 0.86).

## Discussion

To our knowledge this study was the first to directly compare freezing of gait in anxious and non-anxious situations. The primary objective was to use virtual reality to induce anxiety and directly evaluate its influence on freezing of gait in Parkinson's disease. As previously discussed, freezing of gait has been deemed difficult to elicit in experimental settings. Yet, the current experiment led to freezing of gait in 85.7% percent of the freezing population studied and a remarkable 300+ freezing episodes were elicited within this group. To date, this frequency of freezing drawn out in an experimental setting is well beyond the frequency reported in any other freeze-provoking paradigms. Overall, this study provides strong evidence that anxiety can play a causal role in freezing of gait. Freezers spent a significantly greater percentage of each trial frozen when walking across a plank that appeared to be high above a pit (HIGH) compared to walking across the plank located on the ground (LOW). Furthermore, Freezers experienced a significantly greater number of freezing of gait episodes in the HIGH condition compared to the LOW, and also had significantly higher step length variability and step time variability when walking across the HIGH plank. Thus, it is evident from this study that anxiety is an important trigger that may underlie freezing of gait.

It is important to highlight that the anxiety-inducing protocol used in this study effectively manipulated anxiety in all participants. Results showed that all participants reported higher levels of anxiety (using the self-assessment manikins) after walking across the plank high above a deep pit compared to walking across the plank on the ground. In addition, all participants showed more cautious gait (e.g. significantly reduced their velocity, step length and increased their step time variability) when walking across the HIGH plank compared to the LOW. This cautious gait adaptation has been shown in older adults when walking across a HIGH plank in a “real world” setting [37], [38] and demonstrates that both groups in the current study found the virtual environments immersive and realistic enough to elicit more cautious gait and provoke freezing of gait episodes. This provides additional evidence that virtual reality is a very powerful tool for eliciting and studying freezing behaviour in Parkinson's disease in order to better understand the mechanism underlying this phenomenon [17], [24].

### Does anxiety cause freezing of gait?

Although baseline levels of anxiety (i.e. state and trait levels) were not different between groups, anxiety induced during the walking paradigm was significantly amplified in Freezers beyond the level of Non-freezers. This would suggest that in Freezers, goal-oriented movement has the potential to induce greater anxiety, leading to a cautious and potentially maladaptive movement response such as freezing of gait. Thus, in other circumstances such as walking in darkness, or approaching doorways and narrow spaces, the anxiety-driven need for cautious movement might explain the occurrence of freezing in these situations. One might question whether freezing precedes anxiety or if anxiety does in fact lead to a freeze episode. Panic attacks [6] and heart rate increases have been identified [7] prior to and during a freezing episode. However, inferring a causal relationship would be difficult since there was no manipulation of anxiety-inducing conditions. Rather, associations make it ambiguous as to whether panic attacks and autonomic responses provoke freezing of gait or are a reactive response. The current findings support and extend this research, demonstrating that anxiety is in fact a cause of freezing of gait rather than simply a response, since manipulations of anxiety (HIGH and LOW) directly influenced the amount of freezing of gait participants experienced. Spatiotemporal gait changes (i.e. increases in step-to-step variability), which have been previously linked with freezing behaviour, were also increased when anxiety was heightened.

### How does anxiety cause freezing of gait?

Interestingly, increased step-to-step variability also occurs in Non-freezing Parkinson's patients when asked to perform a cognitive dual-task [39], [40]. Thus, it is plausible that increasing limbic “load” may be analogous to a cognitive load in non-freezing Parkinson's participants, in that both overload the capacity for the basal ganglia to process competing inputs. Although there are many models trying to further elucidate the mechanisms behind freezing behaviour [41], the current results fit very well with the cross-talk model, which emphasizes that competing inputs from cognitive, motor and limbic loops all get processed in the striatum, and in instances where there is insufficient dopamine and an overload of information to be processed (e.g. anxiety), freezing of gait occurs [16]. Although there has been support for this model from a cognitive perspective [42]–[45], there has been no study that has tested whether “limbic overload” could produce freezing of gait. The current study effectively demonstrated that “limbic overload” does trigger greater amounts freezing of gait and produces higher step-to-step variability that has been suggested to be conducive to freezing. The cross-talk model also suggests that integrated information processing (i.e. cognitive, motor and limbic) across the basal ganglia circuits is modulated by striatal dopamine; thus when dopamine levels are critically reduced (ex. OFF state) there may be insufficient processing of all information, which result in freezing of gait. In the current study, freezing was quadrupled when participants walked across the HIGH plank in the OFF state, supporting the cross-talk theory. However, anxiety levels did not change with medication at baseline nor during the experimental conditions. In accordance with the cross-talk model, this would suggest that dopaminergic medication increased the capability of the basal ganglia to process the limbic input, rather than reducing the limbic overload in itself [46].

Recently, imaging studies have begun to identify neural correlates associated with freezing behaviour. Although these studies did not focus on inducing anxiety to provoke freezing of gait, it is interesting that decreases in activation were found in the medial prefrontal cortex, left anterior insula and left ventral striatum during motor arrests compared to walking [42]. Although these regions are involved in an array of functions such as the cognitive control network (suggested by the authors), these areas also have a well-established role in emotional processing [47]. A recent review highlighted that nearly 60% of emotional induction studies reported activation of the insula [47], and furthermore the insula has been suggested to participate in evaluation of distressing thoughts and interoceptive emotional responses [48]. Imaging results have also shown that Freezers have significantly less BOLD signal in the bilateral anterior insula and bilateral ventral striatum compared to Non-Freezers during simulated walking in virtual reality with increased cognitive load [45]. Taken together, these results align with the current findings and theoretical framework suggesting that dysfunctional processing of emotional information in the ventral striatum might be one explanation of the current results showing that anxiety increased freezing of gait.

### How do these findings fit within existing models of freezing of gait?

It is important to consider how some models of freezing of gait describe a downstream effect, without addressing the upstream cause. This might be why other models are not able to explain how anxiety or other processes might overload the basal ganglia, leading to increased freezing of gait. For example, the threshold model predicts that a motor deficit can accumulate to the point that reaches a threshold and freezing occurs [49]. This model does not identify a root cause of the initial motor deficit. According to the current results, anxiety might be the key factor that initiates the motor deficit in the first place, and thus this model would be incomplete without the upstream cause having been identified. Similarly, the decoupling model does not identify the initial upstream event that leads to decoupling between preprogrammed and intended motor responses [50]. Thus, in both cases identifying the upstream cause can elucidate why freezing of gait is the resultant behaviour.

In contrast, the cognitive model suggests that freezing of gait is an outcome of a conflict-resolution deficit, specifically exacerbated in situations where response selection and inhibition of unwanted responses are necessary [51]. This model also emphasizes that executive dysfunction might enhance freezing behaviours in these situations. The results from this study do not directly support this model, since response selections were not made during the walking trials. However, one could argue that conflicting signals could arise from limbic or sensory input, and a limited amount of resources (possibly executive dysfunction) might restrict one's ability to resolve this conflict resulting in a freezing episode. If this were the case, this model describes a very similar mechanism as the cross-talk model. A recent cohort study highlighted that persons with PD are unable to modulate step width variability in order to adapt to threatened stability and also ineffectively increase their step width under dual task conditions compared to healthy control participants [52]. In the current study, both groups did not modulate their step width or step width variability when walking across the HIGH plank compared to the LOW, suggesting that cognitive interference may have limited their ability to adapt to threatened stability. Furthermore, Freezers had a smaller step width and reduced step width variability compared to the Non-freezers. Thus, it may be the case that Freezers experienced greater cognitive interference while walking in virtual reality, but since step width and step width variability did not differ between conditions, especially in the Freezers, cognitive interference cannot fully account for the significant increases in freezing of gait when walking across the HIGH plank.

### Limitations and considerations

One limitation of this study was the small number of participants that were able to complete this study OFF their dopaminergic medication. Since our sample was limited, and there were no freezing episodes in the ON state LOW condition, statistical analyses were not performed on the freezing of gait variables between the ON and OFF state. Future research should investigate and confirm our observations of reduced freezing specifically during waking in the anxious environment (HIGH) in the ON state compared to the OFF state. It should also be noted that the participants that were able to complete the study both in ON and OFF states, were much less severe with mild freezing, rather than severe freezing of gait. Therefore, the reported change from OFF to ON in freezing is likely conservative considering these individuals were much higher functioning. Additionally, all participants were unable to see their limbs or body in the virtual environment (since it was dark). Research has suggested that sensory processing is an important contributor to freezing of gait [5] and freezing might be increased when visual feedback about body position is not available. Although this rationale might explain the occurrence of freezing of gait while walking across the plank on the ground (LOW), it cannot explain the massive increases in freezing behaviour experienced when Freezers walked across the plank above the pit, since in both HIGH and LOW conditions visual feedback about body position would have been absent.

## Conclusion

This was the first study to directly compare freezing of gait in anxious and non-anxious situations and showed that virtual reality is a very effective means of inducing anxiety and causing freezing of gait. It was found that Freezers reported significantly higher levels of anxiety compared to Non-freezers. Additionally, over 230 freezing of gait episodes were elicited (in a sample of only 14 Freezers) when walking in the anxious environment (over double that of over ground walking in virtual reality). This study provides strong evidence that anxiety is an important mechanism underlying freezing of gait and suggests that increasing limbic “load” (i.e. anxiety) leads to increased freezing of gait and step-to-step variability. Future studies should investigate whether effectively treating anxiety might reduce the occurrence of freezing of gait and potentially other severe symptoms of Parkinson's disease.
